# Unpacking the relationships between emotions and achievement of EFL learners in China: Engagement as a mediator

**DOI:** 10.3389/fpsyg.2023.1098916

**Published:** 2023-02-20

**Authors:** Haihua Wang, Yingli Wang, Shaojie Li

**Affiliations:** ^1^School of Foreign Languages, Dalian Maritime University, Dalian, China; ^2^Department of English Language and Literature, Korea Maritime and Ocean University, Busan, Republic of Korea

**Keywords:** emotions, foreign language enjoyment, foreign language classroom anxiety, foreign language learning boredom, engagement, English achievement, learners of English as a foreign language, structural equation modeling

## Abstract

Emotions are attracting growing attention in second language acquisition (SLA), especially with the advent of positive psychology (PP). The fundamental role of emotions in affecting learners’ second language (L2) achievement has been well-documented. Evidence also indicates that emotions can significantly influence learners’ L2 learning engagement which profoundly impacts their academic performance. However, the links between emotions, engagement, and L2 achievement remain underexplored. To contribute to this research domain, the present study sought to unpack the relationships between learners’ emotions, such as foreign language enjoyment (FLE), foreign language classroom anxiety (FLCA), and foreign language learning boredom (FLLB), and engagement as well as their English achievement. A total of 907 learners of English as a foreign language (EFL) from a university in China were recruited to complete an online questionnaire. Structural equation modeling (SEM) was performed to test the hypothesized relations among the variables. Results revealed correlations between learners’ FLE, FLCA, and FLLB. Furthermore, learners’ engagement was found to mediate the relationships between their emotions (FLE, FLCA, and FLLB) and English achievement. The findings broaden the nomological network of emotions and engagement in the EFL context, and provide evidence for the mechanism underlying the relationships between emotions, engagement, and achievement, thereby shedding light on EFL teaching and learning at the tertiary level in China.

## 1. Introduction

Learners experience various emotions when they attend class, participate in activities, interact with teachers and peers, or take exams, showing that emotions are ubiquitous in academic and language learning (Pekrun and Linnenbrink-Garcia, 2012; Plonsky et al., 2022). In the past decades, a sizable amount of research on emotions in the field of Second Language Acquisition (SLA) has been carried out, with the main focus on a negative emotion—foreign language classroom anxiety (FLCA). Scholars have correlated this construct with numerous learning variables, including motivation (Hashimoto, 2002; Fathi and Mohammaddokht, 2021), willingness to communicate (WTC; MacIntyre et al., 2002), learner personality traits (Dewaele, 2002), emotional intelligence (Dewaele et al., 2008), learner-related variables (Dewaele, 2013), various measures of language achievement (Shao et al., 2013; Li et al., 2019; Zhang, 2019; Botes et al., 2020), and other emotions (Dewaele and Proietti Ergün, 2020; Dewaele et al., 2022; Li and Han, 2022). However, on the way to learning a foreign language, foreign language enjoyment (FLE) and FLCA were the metaphorical “right and left feet of language learner” (Dewaele and MacIntyre, 2016, p: 215). Therefore, researchers have juxtaposed FLCA with FLE in research.

Recently, with the emergence and flowering of Positive Psychology (PP) in educational psychology, researchers in SLA started to shift their focus from investigating negative emotions to exploring positive affective variables. The role of positive emotions was highlighted, among which FLE was one of the most studied constructs. Scholarly studies on FLE addressed its conceptualization and measurement (Dewaele and MacIntyre, 2014, 2016; Jin and Zhang, 2019), its sources and effects on foreign language learning (Jin and Zhang, 2021; Botes et al., 2022), as well as its linkages with other learner-related variables (Dewaele et al., 2017, 2019a; Li et al., 2021a). Furthermore, the correlations and combined impacts of FLE and FLCA on foreign language achievement have been examined in studies (Dewaele and Alfawzan, 2018; Li et al., 2019; Dewaele and Proietti Ergün, 2020). Apart from FLE and FLCA, another emotion learners most frequently experience in foreign language classrooms is boredom (Pekrun et al., 2010). Despite its popularity in educational psychology for decades, boredom has not received much attention in SLA until recently. Research on foreign language learning boredom (FLLB) concentrated on its conceptualization and measurement (Kruk and Zawodniak, 2017; Li et al., 2021b), as well as antecedents and effects (Kruk and Zawodniak, 2018; Li, 2021; Derakhshan et al., 2021a,b). In language learning, FLE, FLCA, and FLLB could be simultaneously experienced by learners. A holistic view of the complex emotions of foreign language learners can throw light on teachers’ pedagogical practices, and facilitate learners’ language learning to achieve better learning outcomes. Nonetheless, few studies have been undertaken to delve into how FLE, FLCA, and FLLB are associated with achievement (Dewaele et al., 2022; Li and Han, 2022; Li and Wei, 2022), leaving their relationships largely underexplored.

Despite a growing number of studies on emotions in foreign language learning, the nomological network of emotions still needs to be further expanded (Botes et al., 2022). In the field of education and educational psychology, one of the variables that have been evidenced to be the result of emotions is engagement (Oga-Baldwin, 2019), a construct conducive to scholarly success (Pekrun and Linnenbrink-Garcia, 2012; Reschly and Christenson, 2012). Previous studies have demonstrated that engagement was a mediator between learners’ emotions and their academic learning and achievement (Pekrun, 2006; Linnenbrink, 2007). In language education, although studies showed that emotions were associated with engagement (Dewaele and Li, 2021; Mohammad Hosseini et al., 2022), which was recognized as one of the strongest predictors of language achievement (Masgoret and Gardner, 2003), only a handful of studies have expounded on the relationships between emotions, engagement, and achievement in a single study (Dewaele and Li, 2021; Khajavy, 2021; Feng and Hong, 2022).

To fill the research gaps, the present study aimed to unpack the relationships between emotions, engagement, and achievement in language learning by collecting data from Chinese learners of English as a foreign language (EFL) to test a model that hypothesized different emotional variables (FLE, FLCA, FLLB) as predictors of English achievement, with engagement as the mediator. The findings may broaden the nomological network of emotions and engagement in SLA and offers pedagogical implications for EFL teachers and practitioners at the tertiary level.

## 2. Literature review

### 2.1. Emotions

#### 2.1.1. Foreign language classroom anxiety

Since the 1970s, research on affective variables, especially negative emotions, has garnered SLA researchers’ attention (Dewaele et al., 2017). FLCA has been the most studied negative emotion in SLA (MacIntyre, 2017). Horwitz et al. (1986) defined FLCA as “a distinct complex of self-perceptions, beliefs, feelings, and behaviors related to classroom learning arising from the uniqueness of the language learning process” (p: 128), highlighting the multifaceted concept of anxiety. Since the introduction of FLCA in 1986, several scales have been developed to measure it, among which Foreign Language Classroom Anxiety Scale (FLCAS) constructed by Horwitz et al. (1986) has been widely accepted and used by researchers. It is a five-point Likert scale questionnaire comprising 33 items, which has been adapted, shortened, and translated in subsequent studies (Tóth, 2008; Dewaele and MacIntyre, 2014; Dewaele and Al-Saraj, 2015; Li and Wei, 2022).

Adopting various measures of FLCA, researchers have investigated its potential sources, effects, and correlations (Dewaele and MacIntyre, 2014). The existing literature indicated that anxiety of language learners was negatively associated with their motivation (Hashimoto, 2002; Fathi and Mohammaddokht, 2021), their WTC (MacIntyre et al., 2002), and their engagement (Feng and Hong, 2022) in foreign language learning. Also, recent studies have yielded an inverse relationship between FLCA and various language achievement measures (Shao et al., 2013; Li et al., 2019; Zhang, 2019; Botes et al., 2020; Dewaele and Proietti Ergün, 2020; Dewaele et al., 2022; Li and Han, 2022). Shao et al. (2013) explored 510 Chinese EFL students’ emotional intelligence and English classroom learning anxiety, and found negative associations between students’ FLCA and their self-rated English proficiency as well as English achievement measured by CET-4 scores. Dewaele and Proietti Ergün (2020) investigated the relationship between Turkish pupils’ FLCA and their course marks in two foreign languages, Italian and English. The finding exhibited that pupils with high FLCA had lower course marks in both foreign languages. Nevertheless, according to Pekrun (2006) control-value theory, anxiety, as an activating, negative, and achievement-related emotion, can have an ambivalent effect on academic achievement. Therefore, the complex relationship between FLCA and achievement still needs more empirical evidence.

#### 2.1.2. Foreign language enjoyment

With the emergence of PP, researchers in general education gradually shifted their obsession with exploring negative emotions to more positive ones. PP was first introduced in a seminal paper by Seligman and Csikszentmihalyi (2000), who argued the need to focus on the positive aspects of human experience and the reasons for their initiation (MacIntyre, 2021). PP was defined as “the scientific study of what goes right in life, from birth to death and at all stops in between” (Peterson, 2006, p: 4). The two underpinnings for PP are the broaden-and-build theory of positive emotions (Fredrickson, 2001) and the control-value theory of achievement emotions (Pekrun et al., 2002; Pekrun, 2006). The broaden-and-build theory states that positive emotions, including joy, interest, pride, and love, can “broaden people’s momentary thought-action repertoires and build their enduring personal resources” (Fredrickson, 2001, p: 219). On the other hand, based on the control-value theory, achievement emotions can be grouped according to their object focus (activity focus vs. outcome focus), valence (positive vs. negative), and the degree of activation (activating vs. deactivating). However, it was not until recently that PP research “penetrated the mainstream” (Dewaele et al., 2019b) in the field of SLA.

With the popularity of PP in SLA, researchers switched their interest from negative emotions to the positive factors involved in language learning, among which FLE had been one of the most investigated emotions in SLA. Dewaele and MacIntyre (2016) defined FLE as “a complex emotion, capturing interacting dimensions of the challenge and perceived ability that reflect the human drive for success in the face of difficult tasks” (p: 216), which occurs “when people not only meet their needs, but exceed them to accomplish something new or even unexpected” (p: 217). Dewaele and MacIntyre (2014) conducted a pioneering study on the relationship between FLE and FLCA. Based on the FLE scale developed, which comprised 21 items covering various facets of FLE in the foreign language class, a significant and negative correlation was found between FLE and FLCA but with a small amount of shared variance, displaying that they were different emotion dimensions. In addition, levels of FLE were reported to be significantly higher than those of FLCA. This study paved the way for applying PP in SLA (Wang et al., 2021), after which research on FLE in western and eastern contexts flourished.

Studies on FLE have explicated its measurement (Dewaele et al., 2017; Li et al., 2018; Jin and Zhang, 2019; Botes et al., 2021; Jin and Zhang, 2021), and how it is associated with learner-related variables (Dewaele et al., 2019a; Li et al., 2021a). For instance, based on the FLE scale constructed by Dewaele and MacIntyre (2014, 2016), Li et al. (2018) developed the Chinese Version of the FLE Scale (CFLES). By collecting data from 2,078 Chinese students, they conducted a Principal Component Analysis to confirm and validate a new FLE scale containing 11 items with three factors (FLE-Private, FLE-Teacher, and FLE-Atmosphere). The participants reported that their FLE arose through direct teachers’ intervention and indirect peer interaction.

Relevant studies have also explored the relationships between FLE and different measures of foreign language performance. Significantly positive relationships between FLE and both perceived and actual language achievement were found in relevant studies (Piechurska-Kuciel, 2017; Li, 2020; Jin and Zhang, 2021; Botes et al., 2022). Jin and Zhang (2021) collected data from 320 Chinese EFL senior high school students and investigated the dimensions of foreign language classroom enjoyment and their effect on foreign language achievement, and found that the participants’ enjoyment of foreign language learning directly affected mid-term scores, while both enjoyment of teacher support and students support had indirect influences, revealing that FLE impacted foreign language learning in a complex way. A meta-analysis conducted by Botes et al. (2022) suggested a moderate positive correlation between FLE and academic achievement, as well as self-perceived achievement, confirming the significance of FLE in foreign language learning.

Additionally, FLE was also juxtaposed with FLCA since “the combination of positive and negative emotions together is more powerful for influencing teaching practice than looking at them individually” (MacIntyre, 2021, p: 11). Li et al. (2019) examined the correlation between FLE and FLCA, as well as their combined effects on self-perceived English proficiency and actual English achievement of Chinese EFL students. The results revealed a negative correlation between FLE and FLCA, which echoed the previous studies (Dewaele and MacIntyre, 2014, 2016; Dewaele et al., 2016) and was then confirmed in the later study (Dewaele and Proietti Ergün, 2020). Moreover, FLE and FLCA could co-predict self-perceived English proficiency and actual English achievement, with FLCA being the stronger predictor. This result contradicted the findings of Dewaele and Alfawzan (2018) as well as Li and Wei (2022), which showed the positive effect of FLE on performance outweighed that of FLCA. As such, the complex relationships between these two emotions and foreign language performance still need further exploration.

#### 2.1.3. Foreign language learning boredom

Boredom is among the most frequently experienced and potentially devastating academic emotions in the classroom (Pekrun et al., 2010, 2014). It can be defined as “a mild, unpleasant or even painful affective state” that involves “a combination of dissatisfaction, disappointment, annoyance, inattention, lack of motivation to pursue previously set goals and impaired vitality” (Kruk and Zawodniak, 2018, P: 177). Boredom has attracted the interest of researchers in psychology and educational psychology for decades, whereas it was not until relatively recently that boredom received increasing attention in second language (L2) learning and teaching. Chapman (2013) was the first researcher exploring German learners’ and their teachers’ beliefs about boredom. Later, several studies were carried out in the Polish educational context to investigate this negative emotion in terms of changes in the level of boredom (Kruk, 2016a,b), the relationship between boredom experienced by learners and the boredom exhibited in EFL classes (Kruk and Zawodniak, 2017), as well as the experience of boredom in EFL classes (Kruk and Zawodniak, 2018). Other research into boredom concentrated on its impacts on WTC (Zhang et al., 2022), engagement (Dewaele and Li, 2021; Derakhshan et al., 2022), its causes, effects, and solutions in online classes (Derakhshan et al., 2021a,b; Pawlak et al., 2022), together with its conceptualization and measurement (Kruk and Zawodniak, 2017; Li, 2021; Li et al., 2021b). Kruk and Zawodniak (2017) developed the Boredom in Practical English Language Classes Questionnaire (BPELC) to measure this negative emotion in foreign language learning. However, Li et al. (2021b) in their study pointed out the weaknesses of BPELC and they developed the Foreign Language Learning Boredom Scale (FLLBS), a seven-factor scale containing 32 items and exhibiting good psychometric properties, which was adopted in the present study to evaluate the participants’ boredom.

Additionally, some studies juxtaposed FLLB with other emotions, such as FLE and FLCA, to expound their interrelations and influences on foreign language performance. Li and Han (2022) investigated the effects of FLE, FLCA, and FLLB on Chinese EFL learners’ self-perceived and actual achievement in an online learning environment. FLLB was found to have a positive relation with FLCA but a negative association with FLE, and independent negative predictive effects on perceived learning achievement and actual achievement. When entering into the same regression model with FLE and FLCA, FLLB maintained its predictive power on perceived online learning achievement, but failed to directly influence actual test scores. This finding was confirmed in Dewaele et al. (2022) study, showing that FLLB was significantly interrelated with FLE and FLCA, and had no predictive effect on actual achievement when combined with the other two co-predictors. Nevertheless, in the domain of education, the role of boredom as a negative predictor of achievement has been evidenced in quite a few studies (Maroldo, 1986; Pekrun et al., 2009, 2010; Ahmed et al., 2013). The control-value theory also suggested that the effects of boredom can be “detrimental” to academic achievement (Pekrun, 2006). Consequently, FLLB, as an under-investigated emotion in SLA, is in dire need of empirical exploration into its complex relationships with other emotions simultaneously experienced by learners, and its complicated impacts on different measures of foreign language achievement.

### 2.2. Engagement

Engagement, a key contributor to learning and academic success, is about the energy learners spend toward the achievement (Fredricks et al., 2016). As a multifaceted concept describing what and how students think, act, and feel in a classroom setting (Fredricks et al., 2004; Oga-Baldwin, 2019), engagement is conceptualized as being comprised of three dimensions—behavioral, emotional, and cognitive engagement (Fredricks et al., 2004). Behavioral engagement refers to learners’ qualitative behavioral choices in learning (Hiver et al., 2021a), such as their participation, effort, attention, and persistence (Fredricks et al., 2016). Emotional engagement includes the positive and negative affective reactions in the classroom toward teachers, classmates, schools, or school activities (Finn, 1989; Fredricks et al., 2004). Cognitive engagement is conceived as using deep learning strategies and putting effort into comprehending complex ideas (Fredricks et al., 2004). More recently, scholars have proposed additional dimensions to the conceptualization of engagement, such as social engagement (Svalberg, 2009; Wang et al., 2016), agentic engagement (Reeve and Tseng, 2011), and volitional engagement (Filsecker and Kerres, 2014). In language learning research, social engagement has been regarded as a critical dimension of engagement (Hiver et al., 2021a) as it is “essentially linked to interaction and to learners’ initiation and maintenance of it” (Svalberg, 2009, p: 252). To have a holistic understanding of engagement, the dimensions of engagement should be considered together in research instead of focusing on one or two dimensions separately (Zhou et al., 2021). Accordingly, this study delved into four dimensions of engagement, namely behavioral engagement, emotional engagement, cognitive engagement, and social engagement comprehensively.

Engagement, as a significant factor in PP (Wang et al., 2021), has been receiving great attention in educational psychology, on which much research has been conducted regarding its link to better academic achievement (Wang and Holcombe, 2010), self-efficacy (Schunk and Mullen, 2012), achievement goals (Anderman and Patrick, 2012), and emotions (Pekrun and Linnenbrink-Garcia, 2012). Despite its enormous popularity in the educational field, there remains a paucity of research on engagement in SLA. L2 engagement is defined as the extent to which a language learner is involved in doing a language learning task (Hiver et al., 2021b). Studies have connected engagement to foreign language classroom environment (Sulis and Philp, 2021; Mohammad Hosseini et al., 2022), learning and communication mode (Carver et al., 2021), learner-related variables (Mercer and Dörnyei, 2020; Dewale and Li, 2021; Guo, 2021; Derakhshan et al., 2022; Zhao and Yang, 2022), and foreign language achievement (Masgoret and Gardner, 2003; Eren and Rakıcıoğlu-Söylemez, 2020; Kang and Wu, 2022). Nevertheless, the sources and effects of this important construct in foreign language learning still have not been explicated in depth. Previous research in education has proposed the contextual model in which engagement was influenced by the learning environment, such as interpersonal relationships in the classroom, and personal factors like emotions and beliefs. In turn, it affects learners’ future attitudes and achievements (Oga-Baldwin, 2019). Few studies in SLA, specifically in the EFL context, have evidenced the role of L2 engagement as a mediator, especially between emotions and L2 achievement. For example, Khajavy (2021) hypothesized a model in which L2 engagement mediated the relationship between L2 emotions, L2 grit, and L2 reading comprehension in the Iranian EFL context. The finding confirmed the role of L2 engagement as a mediator between perseverance and L2 reading achievement, interest and L2 reading achievement, as well as L2 enjoyment and L2 reading achievement. Feng and Hong (2022) explored the relationship between achievement emotions (FLE and FLCA), behavioral engagement, and self-reported achievement of Chinese EFL learners. The results demonstrated that behavioral engagement mediated the relationship between FLE and self-reported achievement, as well as between FLCA and self-reported achievement. The mediating role of behavioral engagement was also confirmed in Kang and Wu (2022) study, which investigated whether behavioral engagement mediated the academic enjoyment and English achievement of Chinese EFL learners. Therefore, the mediating role of engagement between various emotions and achievement needs further exploration.

Taken together, to our best knowledge, scant research has ever delved into different emotional variables, such as FLE, FLCA, FLLB, L2 engagement, and achievement in a single study, not to mention the mechanism underlying their relationships. To this end, the present study aims to unpack the relationships between emotions and the achievement of EFL learners in China, with engagement as a mediator.

**Figure 1:**
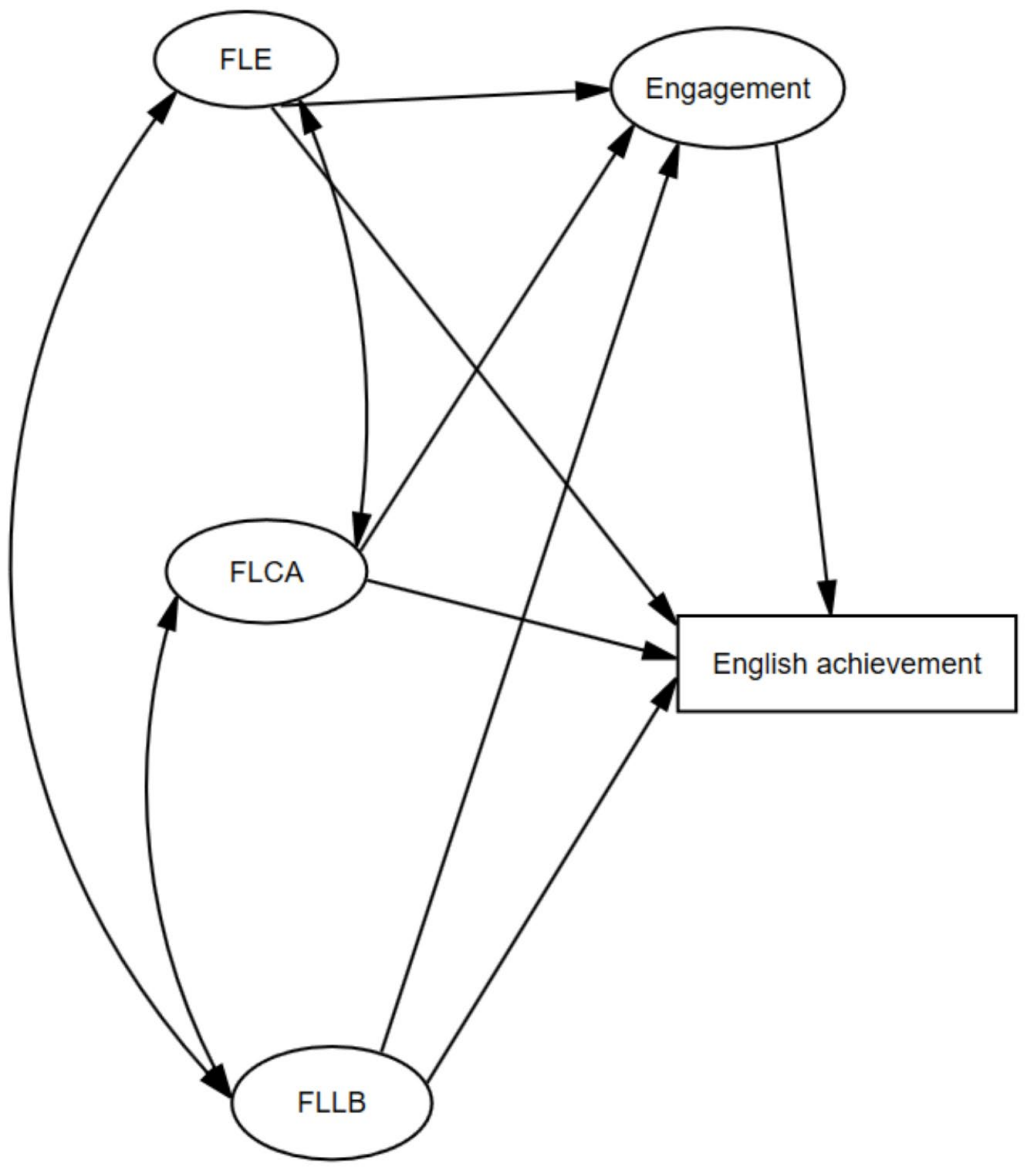
The hypothesized model.

Based on the theoretical and empirical backgrounds of the constructs reviewed above, a structural model of FLE, FLCA, FLLB, L2 engagement, and English achievement was hypothesized. The model and its hypothesized paths are displayed in Figure 1. Given the hypothesized model, the following hypotheses were proposed:

*Hypothesis 1*: FLE, FLCA, and FLLB are correlated significantly.

*Hypothesis 2*: L2 emotions affect L2 engagement significantly.

*Hypothesis 3*: L2 emotions influence English achievement significantly.

*Hypothesis 4*: L2 engagement exerts significant effects on English achievement.

*Hypothesis 5*: L2 engagement mediates L2 emotions and English achievement.

## 3. Methodology

### 3.1. Participants

Convenience sampling was adopted in this study. A total of 921 second-year non-English majors at a university located in northeast China originally participated in the online questionnaire survey. After eliminating 14 questionnaires due to incompleteness, the final sample size was 907. They were from more than 10 majors such as International Economics and Trade, Business Administration, and Logistics Engineering. Among them, 394 were male and 513 were female, with ages ranging from 18 to 23 (*M* = 20.55, *SD* = 0.902). The participants were enrolled in the university after taking the college entrance examination. They were all native Chinese EFL learners with no experience of studying abroad and had learned English for at least 7 years. In China, English teaching at the tertiary level follows the Guidelines for College English Teaching (College Foreign Language Teaching Advisory Committee, 2020). Learners’ skills in English listening, speaking, reading, writing, and translation are mainly cultivated in an English course known as “College English,” a compulsory subject for the participants at this university. They were supposed to take the English course for an average of 3 hours per week for 2 academic years. At the end of the first academic year, they were required to take the College English Test Band 4 (CET-4), a national and standardized test held by the Ministry of Education of the People’s Republic of China to evaluate the undergraduates’ English proficiency. At the time of data collection, all of them had taken the CET-4.

### 3.2. Instruments

The research instrument employed in this study was a composite questionnaire, which was comprised of two major parts: the first part consisted of questions about personal background information (e.g., name, gender, age, and CET-4 scores); the second part was composed of items measuring the participants’ FLE, FLCA, FLLB, and their L2 engagement. All the items in the questionnaire were designed on a five-point Likert scale (1 = never true of me at all to 5 = very true of me). For complete understanding, the items were in Chinese. Before the main administration, the first two authors designed a pilot study and asked their colleagues to carry it out among 212 Chinese EFL learners who made up the peer group for the participants in the main study. The questionnaire administrators were asked to take notes of the questions raised by the respondents when they were filling out the questionnaire, but no question was asked. Based on the item analysis of this pilot study, some modifications were made before the questionnaire was finalized. For example, the item from engagement “In the English course, I enjoyed spending time learning with peers in the class” was eliminated since there was no significance between high score group and the low score group (*p* > 0.05) in its item analysis. A detailed description of the scales used in the questionnaire is as follows.

#### 3.2.1. Foreign language enjoyment

The participants’ enjoyment was measured through an adapted version of the CFLES (Li et al., 2018). It consists of nine items extracted from the CFLES, including three subscales, namely FLE-Private (FLE-P, three items, e.g., I enjoyed learning English.), FLE-Teacher (FLE-T, three items, e.g., The teacher is encouraging. It makes me feel good), and FLE-Atmosphere (FLE-A, three items, e.g., There is a good atmosphere, which makes me feel happy in English class.). All the items were positively phrased. In Li et al. (2018) study, the CFLES was tested among a total of 1,718 Chinese EFL students at the secondary level, and the results showed high reliability with its Cronbach’s alpha reaching 0.83. In the present study, Cronbach’s alpha coefficient was 0.903.

#### 3.2.2. Foreign language classroom anxiety

Anxiety was assessed using the eight-item scale applied in Dewaele and MacIntyre (2014) study, which was developed based on the FLCAS (Horwitz et al., 1986). The eight items investigated the symptoms of anxiety, nervousness, and lack of confidence of the learners. In the scale, six items were phrased to indicate high anxiety, while two reverse-coded items indicated low anxiety. An example is, “Even if I am well prepared for a language class, I feel anxious about it.” In Dewaele and MacIntyre (2014) study, Cronbach’s alpha coefficient was 0.86. In the current study, Cronbach’s alpha coefficient was 0.902.

#### 3.2.3. Foreign language learning boredom

The learners’ boredom was measured by items extracted from the FLLBS (Li et al., 2021b), which contained seven subscales. Three items were used to measure foreign language class boredom (FLLB-FLC, e.g., It is difficult for me to concentrate in the English class.), three for under-challenging task boredom (FLLB-UCT, e.g., I believe an analysis of long text in English is really dreary.), three for homework boredom (FLLB-H, e.g., I get bored of too much English homework.), three for teacher-dislike boredom (FLLB-TD, e.g., The English teacher is uninteresting, so the English class is dull.), three for general learning trait boredom (FLLB-GLT, e.g., Not only learning English, studying is dull in general.), three for PowerPoint presentation boredom (FLLB-PPTP, e.g., Reading from the script in the PPT slides bores me.), and three for over-challenging or meaningless task boredom (FLLB-OCMT; e.g., If I cannot understand classmates’ presentations, I become really bored). As measured by Cronbach’s alpha coefficient, internal consistency was high (0.946).

#### 3.2.4. L2 engagement

L2 engagement was measured from four dimensions through 16 items adapted from Li and Li (2022) questionnaire which was constructed based on the scales developed by Hiver et al. (2020) and Wang et al. (2019). Altogether 12 items were used to assess behavioral engagement (BE, four items, e.g., When I cannot understand in my language class, I stay focused until I do.), emotional engagement (EE, four items, e.g., I enjoy learning new things about languages in class.), and cognitive engagement (CE, four items, e.g., In my language class, I think about different ways to solve a problem.). A total of four items were adapted to evaluate social engagement (SE, four items, e.g., In the English course, I was willing to work with other students, and we could learn from each other.) In the present research, Cronbach’s alpha coefficient for the scale was 0.878.

#### 3.2.5. English achievement

The participants’ English achievement was measured by their scores on the CET-4, a criteria-related, norm-referenced, large-scale English proficiency test administered on behalf of the Ministry of Education of China. The CET-4 corresponds to the standards set in Guidelines for College English Teaching (College Foreign Language Teaching Advisory Committee, 2020), with the aim to assess the English proficiency of the students and provide information for teachers to improve their pedagogical practices. It has strong social impacts and is widely recognized among institutions and employers in Mainland China. The validity and reliability of the CET-4 scores have been established by the previous study (Yang and Weir, 1998), indicating that it is the best fit for measuring the participants’ English achievement.

According to the Syllabus for College English Test—Band Four (National College English Testing Committee, 2016), developed by the National College English Testing Committee, CET-4 is composed of four parts, including listening comprehension, reading comprehension, translation, and writing. After a series of transformation processes, including score weighting, score equating, and score normalization, the reported total test score is 710, with listening comprehension accounting for 35%, reading comprehension 35%, translation 15%, and writing 15%.

### 3.3. Procedure for data collection and analysis

Data collection took place in March 2022. The first author contacted her colleagues individually and asked them to recruit their students as the participants of the present study. Information about the purpose and details of the survey was provided to the teachers and the participants. After obtaining consent, we administered an online questionnaire through Questionnaire Star (a tool for online surveys) to the participants at the beginning of their classes. According to the data provided by Questionnaire Star, the participants took about 15 min on average to complete the questionnaire. As suggested by Dörnyei et al. (2006), there are always a small number of participants who do not take the process seriously in large-scale surveys. Accordingly, before starting the quantitative analyses, we eliminated 14 questionnaires due to incompleteness of the information, which was a low proportion (less than 1.6%) and was therefore considered acceptable.

The quantitative data analyses were performed using the SPSS 23.0 and Amos 21.0 software. Correlations and path analysis were carried out to unpack the relationships between variables. For correlations, effect sizes were used as *r* = 0.25 (small effect size), *r* = 0.40 (medium effect size), and *r* = 0.60 (large effect size; Plonsky and Oswald, 2014). Confirmatory factor analysis (CFA) was run for each construct. The evaluation of the model and CFAs was based on some goodness-of-fit indices. In this study, Chi-square divided by degree of freedom (χ^2^/df), comparative fit index (CFI), Tucker-Lewis index (TLI), root mean square error of approximation (RMSEA), and standardized root mean square residual (SRMR) were applied. To have a fit model, χ^2^/df should be less than 3, CFI and TLI should be above 0.90, and RMSEA and SRMR should be less than 0.08 (Tseng and Schmitt, 2008).

## 4. Results

### 4.1. Validity of the scales

CFA was conducted to assess the measurement model of each construct. Table 1 presents the goodness-of-fit indices for all tested models. Results of the Chi-square test and χ^2^/df indices of FLCA, FLLB, and L2 engagement indicated less-than-adequate fit. Nevertheless, it has been suggested that the Chi-square test may not be accurate with sample sizes over 200 (e.g., Bagozzi and Yi, 1988; Schumacker and Lomax, 1996). As the sample in this study was 907, we adopted the other four fit indices to test the adequacy of each model. The fit indices CFI, TLI, SRMR, and RMSEA showed that all four models fitted the data adequately.

**Table 1:** Goodness of fit indices for the measurement models.

	ꭓ^2^/df	CFI	TLI	SRMR	RMSEA
FLE	2.385	0.994	0.991	0.019	0.039
FLCA	4.845	0.979	0.971	0.026	0.065
FLLB	4.178	0.968	0.960	0.050	0.059
engagement	3.910	0.967	0.959	0.022	0.038

### 4.2. Descriptive statistics and correlations

Descriptive statistics and correlations were calculated for all variables. Table 2 displays descriptive statistics including means, standard deviation, as well as skewness and kurtosis. Concerning the normality of the scales, based on Kline (1998) threshold values, all the absolute values of skewness were lower than 3, and those of kurtosis were lower than 10, demonstrating that the data were normally distributed.

**Table 2:** Descriptive statistics and correlations.

No.	Variables	1	2	3	4	5
1	FLE	1				
2	FLCA	−0.063*	1			
3	FLLB	−0.541**	0.082*	1		
4	Engagement	0.589**	−0.332**	−0.508**	1	
5	English achievement	0.577**	−0.210**	−0.459**	0.609**	1
	Mean	3.380	2.588	2.728	3.487	482.700
	Std. Deviation	0.666	0.740	0.744	0.544	89.512
	Minimum	1.00	1.00	1.00	1.25	221
	Maximum	5.00	4.38	4.81	4.81	651
	Skewness	−0.189	−0.046	0.407	−0.506	−2.007
	Kurtosis	0.331	−0.837	0.694	1.684	5.687

*p < 0.05; **p < 0.01.

Results of Pearson’s correlation analyses suggested that FLE was negatively related to FLCA and FLLB, with a small effect size (*r* = −0.063, *p* < 0.05) and a large effect size (*r* = −0.541, *p* < 0.01), respectively, whereas FLCA was found to have a small and positive relation with FLLB (*r* = 0.082, *p* < 0.05). In terms of the relationship between emotions and engagement, FLE was positively related to engagement, with a large effect size (*r* = 0.589, *p* < 0.01). In contrast, both FLCA and FLLB were negatively related to engagement, with medium (*r* = −0.332, *p* < 0.01) and large (*r* = −0.508, *p* < 0.01) correlations, respectively. Concerning the relationships between emotions and English achievement, a positive relation was obtained between FLE and English achievement, with a large effect size (*r* = 0.577, *p* < 0.01). Meanwhile, both FLCA and FLLB were found to have negative relationship with English achievement, with a small effect size (*r* = −0.210, *p* < 0.01) and a medium effect size (*r* = 0.459, *p* < 0.01), respectively. Finally, results indicated that engagement had a positive and large relation (*r* = 0.609, *p* < 0.01) with English achievement.

### 4.3. SEM analysis

**Figure 2:**
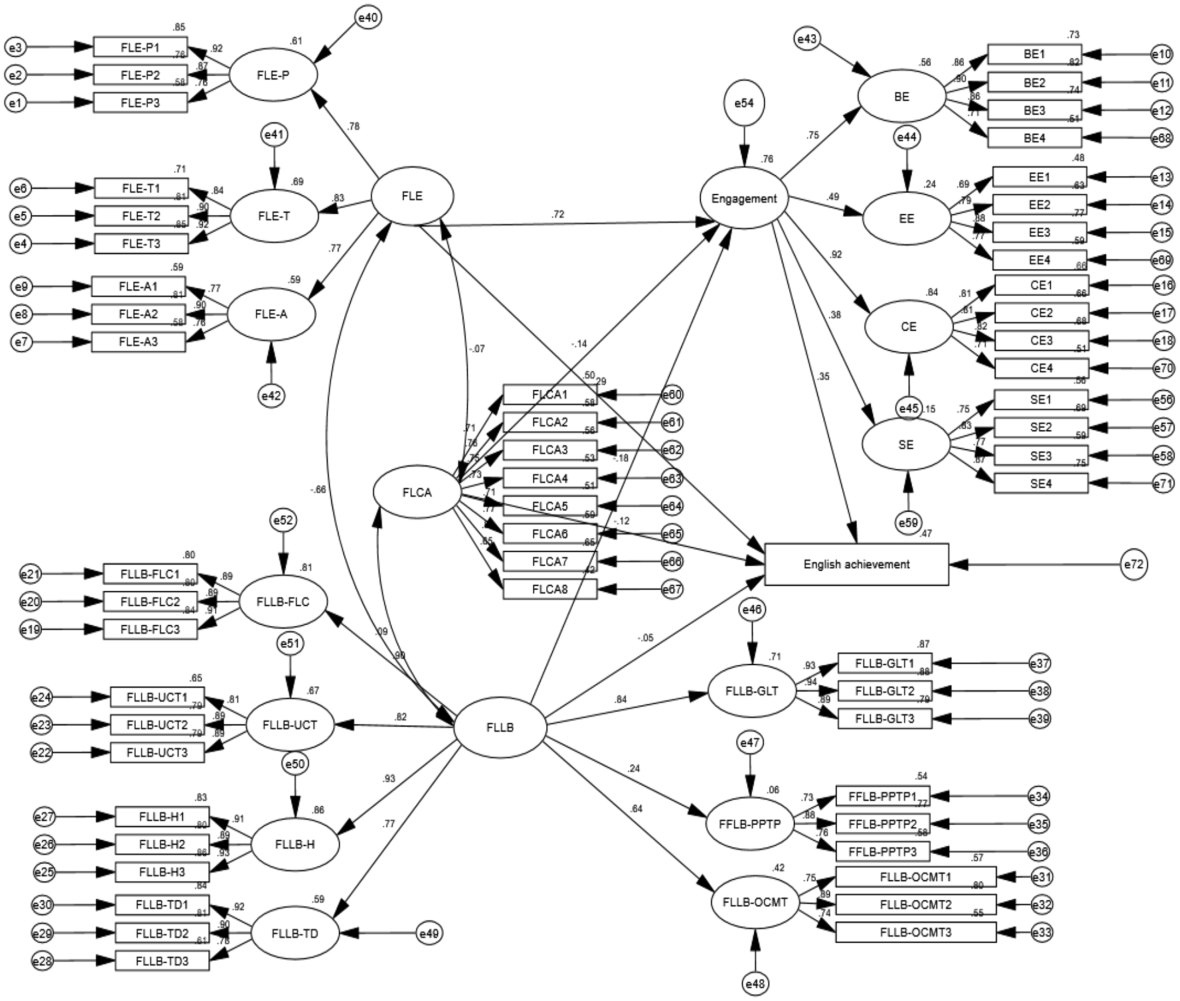
The final model of emotions, engagement, and English achievement.

To unpack the relationships between L2 emotions, L2 engagement, and English achievement, the proposed model was tested with SEM. By using the maximum likelihood method, the overall goodness-of-fit and the standardized coefficient of each path were calculated. The model fit indices in Table 3 showed that the model achieved close fit as CFI (0.918) and TLI (0.913) were both above 0.90. Additionally, both SRMR (0.078) and RMSEA (0.050) were below 0.08. However, χ^2^/df (3.238) was above the maximum limit of 3. Since four of the fit indices met the suggested threshold values, and one was close to the threshold, the model, therefore, fitted the data adequately. Figure 2 depicts the final model for the relationships between L2 emotions, L2 engagement, and English achievement.

**Table 3:** Degree of model fit.

Fitting index	Acceptable range	Measured value
χ^2^/df	<3	3.238
CFI	>0.9	0.918
TLI	>0.9	0.913
SRMR	<0.08	0.078
RMSEA	<0.08	0.050

The standardized estimates for all paths are presented in Table 4. The model accounted for 76.2% of the variance in L2 engagement. Among the three emotions, FLE positively influenced engagement (*β =* 0.717, *p* < 0.05), whereas both FLCA (*β* = −0.136, *p* < 0.001) and FLLB (*β* = −0.185, *p* < 0.05) negatively affected L2 engagement. The role of emotions and engagement in English achievement was also examined. The model accounted for 47.2% of the variance in English achievement. Both FLE (*β* = 0.293, *p* < 0.05) and L2 engagement (*β* = 0.351, *p* < 0.05) exerted positive influences on English achievement. Conversely, FLCA had a negative impact (*β* = −0.121, *p* < 0.05), while FLLB failed to predict English achievement. Bias-corrected bootstrap tests (2,000 times iterations) were performed to test the mediating role of L2 engagement between emotions and English achievement. The 95% confidence interval (CI) showed that L2 engagement mediated the relationships between FLE and English achievement (*β* = 0.251, *p* < 0.05, 95% CI [0.042, 0.437]), FLCA and English achievement (*β* = −0.048, *p* < 0.05, 95% CI [−0.108, −0.009]), as well as FLLB and English achievement (*β* = −0.065, *p* < 0.05, 95% CI [−0.150, −0.009]).

**Table 4:** Unstandardized and standardized path coefficients.

Path relationship	Β	SE	β	Bias-corrected 95%CI		
				Lower	Upper	*P*
FLE→Engagement	0.687	0.059	0.717	0.587	0.822	0.002
FLCA→Engagement	−0.106	0.030	−0.136	−0.200	−0.083	0.000
FLLB→Engagement	−0.119	0.050	−0.185	−0.282	−0.085	0.002
Engagement→English achievement	53.448	0.147	0.351	0.042	0.610	0.030
FLE→English achievement	42.801	0.144	0.293	0.029	0.591	0.027
FLCA→English achievement	−14.415	0.032	−0.121	−0.184	−0.056	0.003
FLLB→English achievement	−5.379	0.060	−0.055	−0.171	0.064	0.365
FLE→Engagement→English achievement	36.742	0.103	0.251	0.042	0.437	0.024
FLCA→Engagement→English achievement	−5.682	0.025	−0.048	−0.108	−0.009	0.018
FLLB→Engagement→English achievement	−6.354	0.035	−0.065	−0.150	−0.009	0.020

## 5. Discussion

This study was intended to unpack the relationships between L2 emotions and English achievement, and whether L2 engagement mediated among the constructs. Questionnaire data were collected to investigate the complex relationships and the underlying mechanism. The results revealed that Chinese EFL learners’ FLE, FLCA, and FLLB predicted their engagement in foreign language learning, which further influenced their English achievement. Despite the corroboration with the findings of the existing literature, the results of the present study displayed some unique features of Chinese EFL learners with regard to their emotional variables, engagement, and achievement.

The findings demonstrated that FLE was negatively correlated with FLCA and FLLB, while FLCA and FLLB were positively interrelated, supporting Hypothesis 1. In other words, learners who experienced more enjoyment in their foreign language learning were less likely to be anxious and bored than those who did not enjoy themselves. Furthermore, learners who reported higher levels of anxiety felt more boredom. In line with the findings of Dewaele et al. (2022) and Li and Han (2022), the obtained results ascertained the interconnections between FLE, FLCA, and FLLB, and confirmed and extended the findings about the negative relationship between FLE and FLCA reported in the previous studies (Dewaele and MacIntyre, 2014, 2016; Dewaele et al., 2016; Li et al., 2019; Dewaele and Proietti Ergün, 2020) and the negative correlation between FLE and FLLB (Dewaele and Li, 2021). Based on the finding that the participants who felt more positive emotions like enjoyment tended to experience fewer negative emotions like anxiety and boredom, it can be argued that positive emotions reduce or neutralize the effects of negative emotions (Dewaele et al., 2022), further evidencing the undoing hypothesis put forward by Fredrickson (2003) in EFL context, which proposes that “positive emotions ‘undo’ the lingering effects of negative emotions” (p: 334).

Results also indicated that all three emotions have significant and direct impacts on L2 engagement, confirming Hypothesis 2. Large, small, and medium correlations were found for the relations between FLE, FLCA, and FLLB with L2 engagement, respectively. Results of path analysis further suggested that FLE was a significant and positive predictor of L2 engagement, whereas FLCA and FLLB negatively predicted L2 engagement. In addition, the R-square (R^2^ = 0.762) revealed that the three emotions “yielded a significant amount of variance” (Zhang et al., 2022) in L2 engagement. Among the three predictors of L2 engagement, FLE was the strongest one, showing the importance of enjoyment in increasing learners’ engagement in language learning. Considering these findings, it can be inferred that when learners enjoyed themselves in language learning, they would be more engaged in foreign language classes. In contrast, they were less likely to participate and involve themselves in foreign language learning when they felt anxious or bored. According to the broaden-and-build theory (Fredrickson, 2001, 2003), such positive emotions as enjoyment can broaden people’s momentary thought-action repertoires and build their enduring personal resources, whereas negative ones narrow people’s thoughts and actions. Accordingly, we can argue that FLE can improve learners’ engagement in learning by enlarging their action repertoire, broadening their momentary mindset, and building their individual resources, while such negative emotions as FLCA and FLLB narrow the same repertoires, thereby decreasing their participation and involvement in language activities. Findings of the present study also provide empirical evidence for the control-value theory (Pekrun, 2006) in the EFL context, which addresses the effects of achievement emotions on student engagement. Regarding the relations found between emotions and engagement, it can be inferred that emotions, specifically enjoyment, play an essential role in engaging learners in the language classroom. The findings, in part, echo those in previous research (Mercer and Dörnyei, 2020; Dewaele and Li, 2021; Guo, 2021; Khajavy, 2021; Derakhshan et al., 2022; Feng and Hong, 2022; Zhao and Yang, 2022).

The third hypothesis that the three emotions significantly influenced English achievement was partly confirmed. Path analysis demonstrated that FLE exerted a positive and significant influence on English achievement. Therefore, learners’ enjoyment of L2 learning improved their English achievement, supporting the findings of previous studies (Piechurska-Kuciel, 2017; Li, 2020; Botes et al., 2021; Jin and Zhang, 2021). This finding also evidences the control-value theory (Pekrun, 2006), which assumes that activating positive emotions like enjoyment benefit academic achievement. Unlike the positive link between FLE and English achievement, FLCA was found to be a negative predictor, indicating that learners who felt anxious about their L2 learning were less likely to perform well in tests, which has been well-documented (Shao et al., 2013; Li et al., 2019; Zhang, 2019; Botes et al., 2020; Dewaele and Proietti Ergün, 2020; Dewaele et al., 2022; Li and Han, 2022). The results also indicated that the effect of FLE (*β* = 0.293, *p* < 0.05) on English achievement outweighed that of FLCA (*β* = −0.121, *p* < 0.05), showing that FLE might be more relevant to learners’ performance in tests, which resonates with the findings of Dewaele and Alfawzan (2018) along with Li and Wei (2022). Surprisingly, FLLB failed to have a significant and direct effect on English achievement when combined with enjoyment and anxiety. This finding is inconsistent with most existing literature that reported a negative impact of boredom on achievement (Pekrun et al., 2009, 2010; Ahmed et al., 2013; Pekrun et al. 2014). This might be because prior research did not involve FLE, FLCA, and FLLB in one model. In the present study, the regression weights of boredom were much lower than those of enjoyment and anxiety as predictors of English achievement, which might result in the non-significant predictive power of FLLB on achievement. Nonetheless, this is not without precedence. Dewaele et al. (2022) also reported that FLLB did not predict achievement when combined with FLE and FLCA. The links between emotions and achievement in this study partly support the findings of Li and Han (2022) research on the relationship between three emotions and English achievement, but are partly inconsistent with theirs. The agreement concerns the significant and negative influence of FLCA on learners’ actual English achievement and the non-significant role of FLLB in learning outcomes. Still, their finding about FLE as a non-significant predictor of achievement differs from the predictive power of FLE displayed here. One possible explanation might be the differences in the learning environment. Li and Han (2022) explored learners’ emotions and their English achievement in the context of online learning during the period of COVID-19 in China. Compared with the traditional learning environment, this change may increase learners’ anxiety, influencing their enjoyment of L2 learning. Nevertheless, the present study did not highlight the learning environment, and the participants took their CET-4 offline. This means that the learning environment might be taken into account when conducting the relevant study.

Hypothesis 4, that L2 engagement had significant effects on English achievement, was also confirmed. Results displayed a positive correlation between L2 engagement and English achievement. Path analysis further showed that L2 engagement exerted a moderate effect (*β* = 0.351, *p* < 0.05) on learning outcomes, being the strongest predictor among all the significant predictors of achievement in this model. This indicated that L2 engagement had its unique variance in L2 achievement, thus demonstrating the importance of L2 engagement in foreign language learning. Based on the findings, it can be inferred that when learners engage in foreign language classroom activities and put effort into language learning, they are more likely to score high on tests. This outcome supports those results that L2 engagement, whether it was treated as a multidimensional concept or a single dimension, had a positive and predictive impact on L2 achievement (Masgoret and Gardner, 2003; Eren and Rakıcıoğlu-Söylemez, 2020; Khajavy, 2021; Feng and Hong, 2022; Kang and Wu, 2022). From a broader perspective, the role of engagement as a critical contributor to learning and academic success was further confirmed, corroborating the findings of the existing literature (Klem and Connell, 2004; Reeve and Lee, 2014; Wang and Fredricks, 2014).

Path analysis also indicated that L2 engagement was a mediator between FLE, FLCA, FLLB, and English achievement, supporting Hypothesis 5. The results revealed that learners’ FLE exerted a significant and positive influence on their engagement in EFL class, which, in turn, positively impacted their English achievement. On the contrary, their FLCA and FLLB negatively affected their engagement in class and then would incur lower scores on tests. The mediating effect of engagement between FLE and English achievement (*β* = 0.251, *p* < 0.05) is larger than those in the other two pathways (*β* = −0.048, *p* < 0.05; *β* = −0.065, *p* < 0.05). The findings partly correspond to Feng and Hong (2022), which reported the mediating role of behavioral engagement between FLE, FLCA, and self-reported achievement, with a larger mediating effect size of engagement between FLE and SRA. Moreover, these results were partly consistent with Kang and Wu (2022) which revealed the mediating role of behavioral engagement between learners’ academic enjoyment and their English achievement. Likewise, the finding that L2 engagement as a mediator between L2 enjoyment and L2 reading comprehension was reported in Khajavy (2021) study. Furthermore, it was noted that FLLB indirectly affected English achievement through L2 engagement, although it failed to affect achievement directly. According to the control-value theory (Pekrun, 2006), for boredom, a negative, deactivating, and activity-related achievement emotion, control, and values refer to the action, but not outcomes. In other words, the attentional focus of a learner experiencing boredom is on the activity of learning, not on their grades on tests. In this regard, we can argue that if learners feel bored in their EFL class, this emotion is less likely to influence their achievement directly, but would decrease their participation and involvement in EFL class activities. The disengagement in learning would, in turn, impact their academic achievement. This is in accord with Pekrun and Linnenbrink-Garcia (2012) emphasis that emotions can influence students’ engagement, which then affects their academic learning and achievement, as has been reported in other scholarship as well (Pekrun, 1992, 2006; Linnenbrink, 2007).

## 6. Conclusion and pedagogical implication

The present study was one of the first attempts to unpack the relationships between learners’ emotions (FLE, FLCA, FLLB), engagement, and their actual English achievement in the EFL context. Through examining whether English achievement was influenced by FLE, FLCA, and FLLB, with the mediating effect of engagement, we found that learners’ FLE and FLCA, respectively, exerted a significantly positive impact and a significantly negative influence on their English achievement both directly and indirectly through the mediation of engagement, whereas FLLB failed to have such an effect. Only through the mediation of engagement could FLLB affect English achievement, indicating that boredom experienced by learners in EFL class did not directly lead to lower achievement. This negative emotion, however, would decrease learners’ participation and involvement in language learning, which, in turn, resulted in lower marks on tests. Another important finding was the significant interconnections between FLE, FLCA, and FLLB. These findings contributed to the literature by uncovering the mechanism underlying the relationships between emotions, engagement, and achievement in the Chinese EFL context, broadened the nomological network of emotions and engagement, and provided empirical evidence to Pekrun’s control-value theory (2006) and Fredrickson (2003) undoing hypothesis in the field of SLA. The current research also highlighted the significance of emotions and engagement in EFL learning, and underscored the importance of learners’ emotions in influencing their engagement.

The findings of the present study had some pedagogical implications for Chinese EFL teachers and practitioners teaching at the tertiary level. The finding that FLE had the largest predictive effects on both engagement and English achievement when combined with FLCA and FLLB suggests that EFL teachers need to take steps to boost learners’ enjoyment in class. For example, they can provide learning materials that learners are interested in and feel capable of dealing with (Pekrun, 2006). In addition, pleasant activities that are controllable and valued positively by learners are also recommended to enhance their FLE, thus increasing their engagement and achievement. Moreover, teacher-related factors, such as emotional support, use of humor, and positive mood, will also play an essential role in affecting learners’ FLE (Dewaele et al., 2019b). But teachers do not need to be excessively anxious about eliminating FLCA and FLLB experienced by learners since positive and negative emotions are like “the right and left feet of language learner” (Dewaele and MacIntyre, 2016, p.215), meaning that a balance will be found between both. Positive emotions like FLE may neutralize the negative effects of the negative emotions according to the results of the correlation analysis. Additionally, given the strongest predictive impact of L2 engagement on English achievement among all the predictors included in the model, teachers are suggested to plan motivating and enjoyable activities to maintain learners’ focus and concentration in EFL class. For instance, teachers can design activities that require a high level of learners’ participation and interaction, instead of being teacher-led (Sulis, 2022). The other finding that engagement mediated FLLB to influence English achievement significantly indicates the negative causal effect of learners’ boredom experiences on their participation in class, which, in turn, impacts their achievement. By implication, teachers should pay due attention to minimizing learners’ boredom in ELF classes. For example, they can design activities or change the learning environment to arouse learners’ interest in language learning as interest can protect against feeling bored (Pekrun et al., 2010).

## 7. Limitations and suggestions for further research

This study has evidenced the relationships between three L2 emotions, L2 engagement, and achievement in the EFL context, offering essential implications. However, there are some limitations to be acknowledged. Firstly, the participants of the present study were recruited from one university in China, thus influencing the generalizability of the results in other settings. Hence, future studies can select larger samples of participants at different proficiency levels from various EFL contexts. Secondly, the present research was cross-sectional, failing to capture dynamic features of emotions and engagement and their impacts on achievement. It is advised that longitudinal studies be carried out to further investigate the dynamic changes of emotions, engagement, and achievement among EFL learners and their reciprocal rather than unidirectional causation across time. Finally, the current study only employed a quantitative method to measure emotions and engagement, which cannot reveal the detailed and dynamic features of the constructs due to individual differences. Further studies are recommended to integrate the quantitative data with qualitative ones, such as data collected from interviews and classroom observation, to achieve a deeper understanding of the in-depth characteristics of the constructs and their relationship.

## Data availability statement

The original contributions presented in the study are included in the article/supplementary material, further inquiries can be directed to the corresponding author.

## Ethics statement

Ethical review and approval were not required for the study on human participants in accordance with the local legislation and institutional requirements. The patients/participants provided their written informed consent to participate in this study.

## Author contributions

HW and YW conceptualized, designed the study, and drafted the manuscript. HW, YW, and SL collected the data and processed the data. All authors contributed to the article and approved the submitted version.

## Funding

This research was supported by the Social Science Foundation of Liaoning Province (Grant no. L21AYY002).

## Conflict of interest

The authors declare that the research was conducted in the absence of any commercial or financial relationships that could be construed as a potential conflict of interest.

## Publisher’s note

All claims expressed in this article are solely those of the authors and do not necessarily represent those of their affiliated organizations, or those of the publisher, the editors and the reviewers. Any product that may be evaluated in this article, or claim that may be made by its manufacturer, is not guaranteed or endorsed by the publisher.

## References

[ref1] Ahmed W. van der Werf G. Kuyper H. Minnaert A. (2013). Emotions, self-regulated learning, and achievement in mathematics: a growth curve analysis. J. Educ. Psychol. 105, 150–161. doi: 10.1037/a0030160

[ref2] Anderman E. M. Patrick H. (2012). “Achievement goal theory, conceptualization ofability/intelligence, and classroom climate” in Handbook of research on student engagement. eds. Christenson S. L. Reschly A. L. Wylie C. (Boston, MA: Springer US), 173–191.

[ref3] Bagozzi R. P. Yi Y. (1988). On the evaluation of structural equation models. J. Acad. Mark. Sci. 16, 74–94. doi: 10.1007/bf02723327

[ref4] Botes E. Dewaele J.-M. Greiff S. (2020). The foreign language classroom anxiety scale and academic achievement: an overview of the prevailing literature and a meta-analysis. Journal for the Psychology of Language Learning 2, 26–56. doi: 10.52598/jpll/2/1/3

[ref5] Botes E. Dewaele J.-M. Greiff S. (2021). The development and validation of the short form of the foreign language enjoyment scale. Mod. Lang. J. 105, 858–876. doi: 10.1111/modl.12741

[ref6] Botes E. Dewaele J.-M. Greiff S. (2022). Taking stock: a meta-analysis of the effects of foreign language enjoyment. Studies in Second Language Learning and Teaching 12, 205–232. doi: 10.14746/ssllt.2022.12.2.3

[ref7] Carver C. Jung D. Gurzynski-Weiss L. (2021). “Examining learner engagement in relationship to learning and communication mode” in Student engagement in the language classroom. eds. Hiver P. Al-Hoorie A. H. Mercer S. (Bristol: Multilingual Matters), 120–142.

[ref8] Chapman K. E. (2013). *Boredom in the German foreign language classroom*. [doctoral dissertation]. [Madison (WI)]: University of Wisconsin-Madison

[ref9] College Foreign Language Teaching Advisory Committee. (2020). Guidelines for college English teaching. Beijing: Higher Education Press.

[ref10] Derakhshan A. Fathi J. Pawlak M. Kruk M. (2022). Classroom social climate, growth language mindset, and student engagement: the mediating role of boredom in learning English as a foreign language. J. Multiling. Multicult. Dev., 1–19. doi: 10.1080/01434632.2022.2099407

[ref11] Derakhshan A. Kruk M. Mehdizadeh M. Pawlak M. (2021a). Activity-induced boredom in online EFL classes. ELT J. 76, 58–68. doi: 10.1093/elt/ccab072

[ref12] Derakhshan A. Kruk M. Mehdizadeh M. Pawlak M. (2021b). Boredom in online classes in the Iranian EFL context: sources and solutions. System 101:102556. doi: 10.1016/j.system.2021.102556

[ref13] Dewaele J.-M. (2002). Psychological and sociodemographic correlates of communicative anxiety in L2 and L3 production. Int. J. Biling. 6, 23–38. doi: 10.1177/13670069020060010201

[ref14] Dewaele J.-M. (2013). The link between foreign language classroom anxiety and psychoticism, extraversion, and neuroticism among adult bi- and multilinguals. Mod. Lang. J. 97, 670–684. doi: 10.1111/j.1540-4781.2013.12036.x

[ref15] Dewaele J.-M. Alfawzan M. (2018). Does the effect of enjoyment outweigh that of anxiety in foreign language performance? Studies in Second Language Learning and Teaching 8, 21–45. doi: 10.14746/ssllt.2018.8.1.2

[ref16] Dewaele J.-M. Al-Saraj T. (2015). Foreign language classroom anxiety of Arab learners of English: the effect of personality, linguistic and sociobiographical variables. Studies in Second Language Learning and Teaching 5, 205–228. doi: 10.14746/ssllt.2015.5.2.2

[ref17] Dewaele J.-M. Botes E. Greiff S. (2022). Sources and effects of foreign language enjoyment, anxiety, and boredom: a structural equation modeling approach. Stud. Second. Lang. Acquis. 1–19, 1–19. doi: 10.1017/s0272263122000328

[ref18] Dewaele J.-M. Chen X. Padilla A. M. Lake J. (2019b). The flowering of positive psychology in foreign language teaching and acquisition research. Front. Psychol. 10:2128. doi: 10.3389/fpsyg.2019.02128, PMID: 31607981 PMC6769100

[ref19] Dewaele J.-M. Li C. (2021). Teacher enthusiasm and students’ social-behavioral learning engagement: the mediating role of student enjoyment and boredom in Chinese EFL classes. Lang. Teach. Res. 25, 922–945. doi: 10.1177/136216882110145

[ref20] Dewaele J.-M. MacIntyre P. D. (2014). The two faces of Janus? Anxiety and enjoyment in the foreign language classroom. Studies in Second Language Learning and Teaching 4, 237–274. doi: 10.14746/ssllt.2014.4.2.5

[ref21] Dewaele J.-M. MacIntyre P. D. (2016). “Foreign language enjoyment and foreign language classroom anxiety: the right and left feet of the language learner” in Positive psychology in SLA. eds. MacIntyre P. Gregersen T. Mercer S. (Bristol: Multilingual Matters), 215–236.

[ref22] Dewaele J.-M. MacIntyre P. Boudreau C. Dewaele L. (2016). Do girls have all the fun? Anxiety and enjoyment in the foreign language classroom. Theory and Practice of Second Language Acquisition 2, 41–63.

[ref23] Dewaele J.-M. Magdalena A. F. Saito K. (2019a). The effect of perception of teacher characteristics on Spanish EFL learners’ anxiety and enjoyment. Mod. Lang. J. 103, 412–427. doi: 10.1111/modl.12555

[ref24] Dewaele J.-M. Petrides K. V. Furnham A. (2008). Effects of trait emotional intelligence and sociobiographical variables on communicative anxiety and foreign language anxiety among adult multilinguals: a review and empirical investigation. Lang. Learn. 58, 911–960. doi: 10.1111/j.1467-9922.2008.00482.x

[ref25] Dewaele J.-M. Proietti Ergün A. L. (2020). How different are the relations between enjoyment, anxiety, attitudes/motivation and course marks in pupils’ Italian and English as foreign languages? Journal of the European Second Language Association 4, 45–57. doi: 10.22599/jesla.65

[ref26] Dewaele J.-M. Witney J. Saito K. Dewaele L. (2017). Foreign language enjoyment and anxiety: the effect of teacher and learner variables. Lang. Teach. Res. 22, 676–697. doi: 10.1177/1362168817692161

[ref27] Dörnyei Z. Csizér K. Németh N. (2006). Motivation, language attitudes and globalisation: a Hungarian perspective. Bristol: Multilingual Matters.

[ref28] Eren A. Rakıcıoğlu-Söylemez A. (2020). Language mindsets, perceived instrumentality, engagement and graded performance in English as a foreign language students. Lang. Teach. Res. 1-31:136216882095840. doi: 10.1177/1362168820958400

[ref29] Fathi J. Mohammaddokht F. (2021). Foreign language enjoyment and anxiety as the correlates of the ideal L2 self in the English as a foreign language context. Front. Psychol. 12:790648. doi: 10.3389/fpsyg.2021.790648, PMID: 34899544 PMC8655844

[ref30] Feng E. Hong G. (2022). Engagement mediates the relationship between emotion and achievement of Chinese EFL learners. Front. Psychol. 13:895594. doi: 10.3389/fpsyg.2022.895594, PMID: 35865704 PMC9295811

[ref31] Filsecker M. Kerres M. (2014). Engagement as a volitional construct. Simul. Gaming 45, 450–470. doi: 10.1177/1046878114553569

[ref32] Finn J. D. (1989). Withdrawing from school. Rev. Educ. Res. 59, 117–142. doi: 10.3102/00346543059002117

[ref33] Fredricks J. A. Blumenfeld P. C. Paris A. H. (2004). School engagement: potential of the concept, state of the evidence. Rev. Educ. Res. 74, 59–109. doi: 10.3102/00346543074001059

[ref34] Fredricks J. A. Filsecker M. Lawson M. A. (2016). Student engagement, context, and adjustment: addressing definitional, measurement, and methodological issues. Learn. Instr. 43, 1–4. doi: 10.1016/j.learninstruc.2016.02.002

[ref35] Fredrickson B. L. (2001). The role of positive emotions in positive psychology: the broaden-and-build theory of positive emotions. Am. Psychol. 56, 218–226. doi: 10.1037//0003-066x.56.3.218 11315248 PMC3122271

[ref36] Fredrickson B. L. (2003). The value of positive emotions. Am. Sci. 91, 330–335. doi: 10.1511/2003.4.330

[ref37] Guo Y. (2021). Exploring the dynamic interplay between foreign language enjoyment and learner engagement with regard to EFL achievement and absenteeism: a sequential mixed methods study. Front. Psychol. 12:766058. doi: 10.3389/fpsyg.2021.766058, PMID: 34721246 PMC8554116

[ref38] Hashimoto Y. (2002). Motivation and willingness to communicate as predictors of reported L2 use: the Japanese context. Second Language Studies 20, 29–70.

[ref39] Hiver P. Al-Hoorie A. H. Mercer S. (2021a). Student engagement in the language classroom. Bristol: Channel View Publications.

[ref40] Hiver P. Al-Hoorie A. H. Vitta J. P. Wu J. (2021b). Engagement in language learning: a systematic review of 20 years of research methods and definitions. Lang. Teach. Res. 1–30:136216882110012. doi: 10.1177/13621688211001289

[ref41] Hiver P. Zhou S. Tahmouresi S. Sang Y. Papi M. (2020). Why stories matter: exploring learner engagement and metacognition through narratives of the L2 learning experience. System 91:102260. doi: 10.1016/j.system.2020.102260

[ref42] Horwitz E. K. Horwitz M. B. Cope J. (1986). Foreign language classroom anxiety. Mod. Lang. J. 70, 125–132. doi: 10.1111/j.1540-4781.1986.tb05256.x

[ref43] Jin Y. Zhang L. J. (2019). A comparative study of two scales for foreign language classroom enjoyment. Percept. Mot. Skills 126, 1024–1041. doi: 10.1177/0031512519864471, PMID: 31345113

[ref44] Jin Y. Zhang L. J. (2021). The dimensions of foreign language classroom enjoyment and their effect on foreign language achievement. Int. J. Biling. Educ. Biling. 24, 948–962. doi: 10.1080/13670050.2018.1526253

[ref45] Kang X. Wu Y. (2022). Academic enjoyment, behavioral engagement, self-concept, organizational strategy and achievement in EFL setting: a multiple mediation analysis. PLoS One 17:e0267405. doi: 10.1371/journal.pone.0267405, PMID: 35486654 PMC9053798

[ref46] Khajavy G. (2021). “Modeling the relations between foreign language engagement, emotions, grit and reading achievement” in Student engagement in the language classroom. eds. Hiver P. Al-Hoorie A. Mercer S. (Bristol: Multilingual Matters), 241–259.

[ref47] Klem A. M. Connell J. P. (2004). Relationships matter: linking teacher support to student engagement and achievement. J. Sch. Health 74, 262–273. doi: 10.1111/j.1746-1561.2004.tb08283.x, PMID: 15493703

[ref48] Kline R. B. (1998). Principles and practice of structural equation modeling. New York: Guilford Press.

[ref49] Kruk M. (2016a). “Investigating the changing nature of boredom in the English language classroom: results of a study” in Nowy wymiarfilologii. eds. Dlutek A. Pietrzak D. (Plock: Wydawnictwo NaukowePanstwowej Wyzszej Szkoly Zawodowej w Plocku), 252–263.

[ref50] Kruk M. (2016b). Variations in motivation, anxiety and boredom in learning English in second life. The EUROCALL Review 24, 25–39. doi: 10.4995/eurocall.2016.5693

[ref51] Kruk M. Zawodniak J. (2017). Nuda a praktyczna nauka języka angielskiego. Neofilolog 49, 115–131. doi: 10.14746/n.2017.49.1.07

[ref52] Kruk M. Zawodniak J. (2018). “Boredom in practical English language classes: insights from interview data” in Interdisciplinary views on the English language, literature and culture. eds. Szymanski L. Zawodniak J. Lobodziec A. Smoluk M. (Poland: Uniwersytet Zielonogórski), 177–191.

[ref53] Li C. (2020). A positive psychology perspective on Chinese EFL students’ trait emotional intelligence, foreign language enjoyment and EFL learning achievement. J. Multiling. Multicult. Dev. 41, 246–263. doi: 10.1080/01434632.2019.1614187

[ref54] Li C. (2021). A control–value theory approach to boredom in English classes among university students in China. Mod. Lang. J. 105, 317–334. doi: 10.1111/modl.12693

[ref58] Li C. Dewaele J.-M. Hu Y. (2021b). Foreign language learning boredom: conceptualization and measurement. Applied Linguistics Review 0, 1–27. doi: 10.1515/applirev-2020-0124

[ref56] Li C. Dewaele J.-M. Jiang G. (2019). The complex relationship between classroom emotions and EFL achievement in China. Applied Linguistics Review 11, 485–510. doi: 10.1515/applirev-2018-0043

[ref57] Li C. Han Y. (2022). The predictive effects of foreign language anxiety, enjoyment, and boredom on learning outcomes in online English classrooms. Modern Foreign Languages «现代外语» 45, 207–219.

[ref55] Li C. Huang J. Li B. (2021a). The predictive effects of classroom environment and trait emotional intelligence on foreign language enjoyment and anxiety. System 96:102393. doi: 10.1016/j.system.2020.102393

[ref59] Li C. Jiang G. Dewaele J.-M. (2018). Understanding Chinese high school students’ foreign language enjoyment: validation of the Chinese version of the foreign language enjoyment scale. System 76, 183–196. doi: 10.1016/j.system.2018.06.004

[ref60] Li Z. Li J. (2022). Using the flipped classroom to promote learner engagement for the sustainable development of language skills: a mixed-methods study. Sustainability 14:5983. doi: 10.3390/su14105983

[ref61] Li C. Wei L. (2022). Anxiety, enjoyment, and boredom in language learning amongst junior secondary students in rural China: how do they contribute to L2 achievement? Stud. Second. Lang. Acquis. 1–16, 1–16. doi: 10.1017/S0272263122000031

[ref62] Linnenbrink E. A. (2007). “The role of affect in student learning: a multi-dimensional approach to considering the interaction of affect, motivation, and engagement” in Emotion in education. eds. Schutz P. A. Pekrun R. (Burlington: Academic Press), 107–124.

[ref63] MacIntyre P. D. (2017). “An overview of language anxiety research and trends in its development” in New insights into language anxiety: Theory, research and educational implications. eds. Gkonou C. Daubney M. Dewaele J. (Bristol: Multilingual Matters), 11–30.

[ref64] MacIntyre P. D. (2021). “Exploring applications of positive psychology in SLA” in Positive psychology in second and foreign language education. eds. Budzinska K. Majchrzak O. (Cham: Springer), 3–17.

[ref65] MacIntyre P. D. Baker S. C. Clément R. Donovan L. A. (2002). Sex and age effects on willingness to communicate, anxiety, perceived competence, and L2 motivation among junior high school French immersion students. Lang. Learn. 52, 537–564. doi: 10.1111/1467-9922.00194

[ref66] Maroldo G. K. (1986). Shyness, boredom, and grade point average among college students. Psychol. Rep. 59, 395–398. doi: 10.2466/pr0.1986.59.2.395, PMID: 3786614

[ref67] Masgoret A. M. Gardner R. C. (2003). Attitudes, motivation, and second language learning: a meta-analysis of studies conducted by Gardner and associates. Lang. Learn. 53, 167–210. doi: 10.1111/1467-9922.00227

[ref68] Mercer S. Dörnyei Z. (2020). Engaging language learners in contemporary classrooms. Cambridge: Cambridge University Press.

[ref69] Mohammad Hosseini H. Fathi J. Derakhshesh A. Mehraein S. (2022). A model of classroom social climate, foreign language enjoyment, and student engagement among English as a foreign language learners. Front. Psychol. 13:933842. doi: 10.3389/fpsyg.2022.933842, PMID: 36059776 PMC9428561

[ref70] National College English Testing Committee. (2016). Syllabus for college English test–band four. Shanghai: Shanghai Jiao Tong University Press.

[ref71] Oga-Baldwin W. L. Q. (2019). Acting, thinking, feeling, making, collaborating: the engagement process in foreign language learning. System 86:102128. doi: 10.1016/j.system.2019.102128

[ref72] Pawlak M. Derakhshan A. Mehdizadeh M. Kruk M. (2022). Boredom in online English language classes: mediating variables and coping strategies. Lang. Teach. Res. 1-26:136216882110649. doi: 10.1177/13621688211064944

[ref73] Pekrun R. (1992). The impact of emotions on learning and achievement: towards a theory of cognitive/motivational mediators. Appl. Psychol. 41, 359–376. doi: 10.1111/j.1464-0597.1992.tb00712.x

[ref74] Pekrun R. (2006). The control-value theory of achievement emotions: assumptions, corollaries, and implications for educational research and practice. Educ. Psychol. Rev. 18, 315–341. doi: 10.1007/s10648-006-9029-9

[ref75] Pekrun R. Elliot A. J. Maier M. A. (2009). Achievement goals and achievement emotions: testing a model of their joint relations with academic performance. J. Educ. Psychol. 101, 115–135. doi: 10.1037/a0013383

[ref76] Pekrun R. Goetz T. Daniels L. M. Stupnisky R. H. Perry R. P. (2010). Boredom in achievement settings: exploring control–value antecedents and performance outcomes of a neglected emotion. J. Educ. Psychol. 102, 531–549. doi: 10.1037/a0019243

[ref77] Pekrun R. Goetz T. Titz W. Perry R. P. (2002). Academic emotions in students’ self-regulated learning and achievement: a program of qualitative and quantitative research. Educ. Psychol. 37, 91–105. doi: 10.1207/S15326985EP3702_4

[ref78] Pekrun R. Hall N. C. Goetz T. Perry R. P. (2014). Boredom and academic achievement: testing a model of reciprocal causation. J. Educ. Psychol. 106, 696–710. doi: 10.1037/a0036006

[ref79] Pekrun R. Linnenbrink-Garcia L. (2012). “Academic emotions and student engagement” in Handbook of research on student engagement. eds. Christenson S. L. Reschly A. L. Wylie C. (Boston, MA: Springer US), 259–282.

[ref80] Peterson C. (2006). A primer in positive psychology. New York: Oxford University Press.

[ref82] Piechurska-Kuciel E. (2017). “L2 or L3? Foreign language enjoyment and proficiency” in Multiculturalism, multilingualism and the self. eds. Gabrys-Barker D. Galajda D. Wojtaszek A. Zakrajewski P. (Cham: Springer), 97–111.

[ref83] Plonsky L. Oswald F. L. (2014). How big is ‘big’? Interpreting effect sizes in L2 research. Lang. Learn. 64, 878–912. doi: 10.1111/lang.12079

[ref84] Plonsky L. Sudina E. Teimouri Y. (2022). Language learning and emotion. Lang. Teach. 55, 346–362. doi: 10.1017/s0261444821000434

[ref85] Reeve J. Lee W. (2014). Students’ classroom engagement produces longitudinal changes in classroom motivation. J. Educ. Psychol. 106, 527–540. doi: 10.1037/a0034934

[ref86] Reeve J. Tseng C.-M. (2011). Agency as a fourth aspect of students’ engagement during learning activities. Contemp. Educ. Psychol. 36, 257–267. doi: 10.1016/j.cedpsych.2011.05.002

[ref87] Reschly A. Christenson S. L. (2012). “Jingle, jangle, and conceptual haziness: evolution and future directions of the engagement construct” in Handbook of research on student engagement. eds. Christenson S. L. Reschly A. L. Wylie C. (Boston, MA: Springer), 3–20.

[ref88] Schumacker R. E. Lomax R. G.. (1996). A Beginner’s guide to structural equation modelling. Mahwah, NJ: Lawrence Erlbaum.

[ref89] Schunk D. H. Mullen C. A. (2012). “Self-efficacy as an engaged learner” in Handbook of research on student engagement. eds. Christenson S. L. Reschly A. L. Wylie C. (Boston, MA: Springer US), 219–236.

[ref90] Seligman M. E. P. Csikszentmihalyi M. (2000). Positive psychology: an introduction. Am. Psychol. 55, 5–14. doi: 10.1037/0003-066X.55.1.5 11392865

[ref91] Shao K. Yu W. Ji Z. (2013). An exploration of Chinese EFL students’ emotional intelligence and foreign language anxiety. Mod. Lang. J. 97, 917–929. doi: 10.1111/j.1540-4781.2013.12042.x

[ref92] Sulis G. (2022). Engagement in the foreign language classroom: micro and macro perspectives. System 110:102902. doi: 10.1016/j.system.2022.102902

[ref93] Sulis G. Philp J. (2021). “Exploring connections between classroom environment and engagement in the foreign language classroom” in Student engagement in the language classroom. eds. Hiver P. Al-Hoorie A. H. Mercer S. (Bristol: Multilingual Matters), 101–119.

[ref94] Svalberg A. M.-L. (2009). Engagement with language: interrogating a construct. Lang. Aware. 18, 242–258. doi: 10.1080/09658410903197264

[ref95] Tóth Z. (2008). A foreign language anxiety scale for Hungarian learners of English. Working papers in language. Pedagogy 2, 55–78.

[ref400] Tseng W.-T. Schmitt N. (2008). Toward a model of motivated vocabulary learning: a structural equation modeling approach. Language Learning 58:357–400. doi: 10.1111/j.1467-9922.2008.00444.x, PMID: 34489835

[ref96] Wang Y. Derakhshan A. Zhang L. J. (2021). Researching and practicing positive psychology in second/foreign language learning and teaching: the past, current status and future directions. Front. Psychol. 12:731721. doi: 10.3389/fpsyg.2021.731721, PMID: 34489835 PMC8417049

[ref97] Wang M. T. Fredricks J. A. (2014). The reciprocal links between school engagement, youth problem behaviors, and school dropout during adolescence. Child Dev. 85, 722–737. doi: 10.1111/cdev.12138, PMID: 23895361 PMC3815520

[ref98] Wang M. T. Fredricks J. A. Ye F. Hofkens T. L. Linn J. S. (2016). The math and science engagement scales: scale development, validation, and psychometric properties. Learn. Instr. 43, 16–26. doi: 10.1016/j.learninstruc.2016.01.008

[ref99] Wang M. T. Fredricks J. A. Ye F. Hofkens T. L. Linn J. S. (2019). Conceptualization and assessment of adolescents’ engagement and disengagement in school: a multidimensional school engagement scale. Eur. J. Psychol. Assess. 35, 592–606. doi: 10.1027/1015-5759/a000431

[ref100] Wang M. T. Holcombe R. (2010). Adolescents’ perceptions of school environment, engagement, and academic achievement in middle school. Am. Educ. Res. J. 47, 633–662. doi: 10.3102/0002831209361209

[ref101] Yang H. Weir C. J. (1998). The CET validation study. Shanghai: Shanghai Foreign Language Education Press.

[ref102] Zhang X. (2019). Foreign language anxiety and foreign language performance: a meta-analysis. Mod. Lang. J. 103, 763–781. doi: 10.1111/modl.12590

[ref103] Zhang L. J. Saeedian A. Fathi J. (2022). Testing a model of growth mindset, ideal L2 self, boredom, and WTC in an EFL context. J. Multiling. Multicult. Dev. 1–16, 1–16. doi: 10.1080/01434632.2022.2100893

[ref104] Zhao Y. Yang L. (2022). Examining the relationship between perceived teacher support and students’ academic engagement in foreign language learning: enjoyment and boredom as mediators. Front. Psychol. 13:987554. doi: 10.3389/fpsyg.2022.987554, PMID: 36204761 PMC9530908

[ref105] Zhou S. Hiver P. Al-Hoorie A. H. (2021). “Measuring L2 engagement: a review of issues and applications” in Student engagement in the language classroom. eds. Hiver P. Al-Hoorie A. H. Mercer S. (Bristol: Multilingual Matters), 75–98.

